# Fluorine‐Doping of Mesoporous TiO_2_ Enables Efficient Defect Passivation for High‐Efficiency and Stable Perovskite Solar Cells

**DOI:** 10.1002/advs.202524360

**Published:** 2026-04-02

**Authors:** Hye W. Chun, Sang Yeon Lee, Sang Eun Yoon, Gyeong G. Jeon, Veera Murugan Arivunithi, So Jeong Shin, Min Jun Choi, Byeongsu Kim, Min‐Ho Lee, Dohyeon Jeon, Taekyeong Kim, Jung‐Yong Lee, Jong H. Kim

**Affiliations:** ^1^ Department of Molecular Science and Technology Ajou University Suwon Republic of Korea; ^2^ School of Electrical Engineering Korea Advanced Institute of Science and Technology (KAIST) Daejeon Republic of Korea; ^3^ School of Electrical and Electronic Engineering University of Ulsan Ulsan Republic of Korea; ^4^ Department of Physics Hankuk University of Foreign Studies Yongin Republic of Korea

**Keywords:** fluorination, perovskite solar cells, power conversion efficiency, stability, fluorine doping, trap passivation

## Abstract

Mesoporous titanium dioxide (m‐TiO_2_) is widely used as an electron transport layer (ETL) in perovskite solar cells (PSCs), but its large surface area introduces oxygen‐vacancy‐related trap states that accelerate nonradiative recombination, particularly under low‐intensity illumination. Here, we report a sulfur hexafluoride (SF_6_) reactive ion etching (RIE) plasma fluorination strategy that passivates oxygen vacancies in m‐TiO_2_ through the formation of Ti─F bonds. X‐ray photoelectron spectroscopy and electron‐only‐device characterizations confirm a reduced trap density after fluorination. The fluorinated m‐TiO_2_ (F‐doped TiO_2_) exhibits increased electrical conductivity, an upward Fermi‐level shift, and enhanced surface hydrophobicity, which collectively facilitate the high‐quality growth of FAPbI_3_ films with larger grains, improved crystallinity, and reduced microstrain. PSCs incorporating F‐doped TiO_2_ achieve PCEs of 25.13% under one‐sun illumination and 36.19% under 1000‐lux LED illumination. In addition, wide‐bandgap perovskite devices also show improved performance, demonstrating the generality of the fluorination strategy. Unencapsulated devices retain 80% of their initial efficiency after 2000 h of ambient storage, confirming improved long‐term stability. These results establish SF_6_ RIE plasma fluorination of TiO_2_ as a simple and broadly applicable route to enhancing both efficiency and stability in PSCs across diverse operating conditions.

## Graphical abstract

We introduce an SF_6_ RIE plasma fluorination method for mesoporous TiO_2_, suppressing oxygen vacancies and improving charge extraction. The fluorinated TiO_2_ promotes uniform FAPbI_3_ film formation and enhances device performance under both one‐sun and indoor illumination. Improved moisture resistance further supports long‐term operational stability, and this approach provides a broadly applicable strategy for high‐performance PSCs.

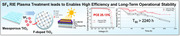

## 1 Introduction

Organic–inorganic hybrid perovskite solar cells (PSCs) have emerged as highly promising photovoltaic technologies owing to their exceptional optoelectronic properties, such as long carrier diffusion lengths, high absorption coefficient in the visible range, and compatibility with low‐temperature, solution‐processed fabrication [1]. Over the past decade, continuous advances in perovskite composition engineering and interface optimization have enabled the power conversion efficiency (PCE) of PSCs to rise from 3.8% in 2009 to beyond 27% [2]. Within this remarkable progress, the choice and engineering of charge‐transport layers have become critical factors governing not only device efficiency but also operational stability and reproducibility of PSCs.

PSCs are typically classified into two major architectures: the p‐i‐n and n‐i‐p structures. To improve photovoltaic performances of p‐i‐n PSCs, diverse strategies for ETL optimization have been explored, ranging from organic semiconductor functionalization to the elemental doping of inorganic components within the ETL to enhance electron extraction and moisture resistance [3, 4]. On the other hand, in n‐i‐p architecture, metal oxides such as titanium dioxide (TiO_2_) or tin dioxide (SnO_2_) are commonly employed as the ETL, which provides high transparency, n‐type conductivity, and robust chemical stability.

Among these metal oxide ETL materials, TiO_2_ has been extensively employed as an ETL owing to its high optical transparency, chemical stability, and favorable energy level alignment with the perovskite absorber [5]. In particular, the incorporation of a mesoporous TiO_2_ (m‐TiO_2_) scaffold atop a compact TiO_2_ (c‐TiO_2_) layer provides an enlarged interface that facilitates charge extraction from perovskite, improving charge photocurrents. However, m‐TiO_2_ intrinsically exhibits limited electron mobility and contains a substantial density of lattice defects, particularly oxygen vacancies [6]. These defect states serve as charge‐trapping centers that accelerate interfacial non‐radiative recombination, reduce the electron transport efficiency within the scaffold, which ultimately constrains the achievable device performance.

To address these limitations, elemental doping of the TiO_2_ has been extensively investigated to enhance its electrical conductivity, mitigate defect‐induced recombination, and modulate the energy levels. In this regard, a variety of dopants, including lithium (Li), tin (Sn), cobalt (Co), and boron (B), have been incorporated into TiO_2_ [7, 8, 9]. Among them, fluorine (F) is particularly attractive because of its extremely high electronegativity (3.98) [10] and strong affinity for titanium (Ti) [11]. F withdraws excess electron density from Ti sites and neutralizes Ti^3+^ states associated with oxygen vacancies through the formation of robust Ti─F bonds, thereby eliminating deep trap states. Additionally, the bond strength of Ti─F (581 kJ mol^−1^) greatly exceeds that of Ti─O (478 kJ mol^−1^) [12], which suppresses the generation of oxygen vacancies while the small atomic radius and low polarizability of F enable lattice incorporation without inducing structural distortion [13]. These combined merits make fluorine doping (F‐doping) an effective strategy for defect passivation and energy‐level modulation in TiO_2_ [14, 15, 16, 17].

Recent studies have validated the effectiveness of F‐doping for TiO_2_ in suppressing defect states and improving device performance. Deng et al. [18]. employed hydrofluoric acid (HF) solution treatment to simultaneously remove surface hydroxyl groups and substitute F at oxygen‐vacancy sites, resulting in reduced trap‐state density and enhanced charge extraction; their PSCs with HF‐treated TiO_2_ showed an improved PCE from 21.76% to 22.86% and maintained over 90% of their initial efficiency even after 1000 h of maximum‐power‐point tracking. Similarly, Hu et al. [15]. introduced F through an in situ TiF_4_ treatment, forming Ti─F bonds that suppress the photocatalytic activity of TiO_2_ under UV irradiation. PSCs employing TiF_4_‐treated TiO_2_ achieved an efficiency enhancement from 19.17% to 22.68%, and preserved 68% of their initial PCE after 26 h of continuous 365 nm UV exposure. These studies collectively demonstrate that fluorine incorporation effectively passivates trap states and improves stability. However, the reported F‐doping methods primarily rely on solution‐based processes using F‐containing precursors such as HF and TiF_4_. Owing to their high sensitivity to ambient moisture and oxygen, these precursors readily undergo hydrolysis, producing undesirable chemical by‐products [19, 20, 21, 22, 23]. Consequently, the fluorination process becomes highly process‐dependent, which hampers precise control over dopant incorporation and makes it challenging to achieve uniform doping and high reproducibility. This limited controllability ultimately causes pronounced device‐to‐device performance variability and compromises the operational stability of PSCs.

Herein, we introduce a plasma‐based fluorination strategy that directly incorporates elemental F into TiO_2_ through a sulfur hexafluoride (SF_6_) reactive ion etching (RIE) process. The SF_6_ plasma generates highly reactive F radicals, which preferentially occupy oxygen‐vacancy sites and form robust Ti─F bonds, thereby effectively passivating oxygen vacancies and associated Ti^3+^ defect states in TiO_2_. The fluorinated TiO_2_ (F‐doped TiO_2_) films exhibit substantially increased electrical conductivity relative to the undoped counterparts, indicating suppressed trap‐assisted charge scattering and improved electron transport. The PSCs based on F‐doped TiO_2_ achieved highly enhanced PCEs up to 25.13% under one‐sun illumination and 36.19% under 1000‐lux LED illumination, outperforming the undoped TiO_2_‐based devices (23.23% and 32.87%, respectively). Distinct enhancements in the open‐circuit voltage (*V*_oc_) and fill factor (FF) confirmed the substantial reduction of non‐radiative recombination by F‐doped TiO_2_. Furthermore, the F‐doped TiO_2_ devices retained 80% of their initial efficiency even after 2240 h of ambient storage without encapsulation, demonstrating significantly improved operational stability. These results indicate that SF_6_ RIE plasma fluorination provides a direct, uniform, and controllable route for F incorporation into TiO_2_ to effectively passivate defects and improve the efficiency of PSCs.

## 2 Result and Discussion

### 2.1 Formation and Characterization of F‐Doped TiO_2_

**FIGURE 1:**
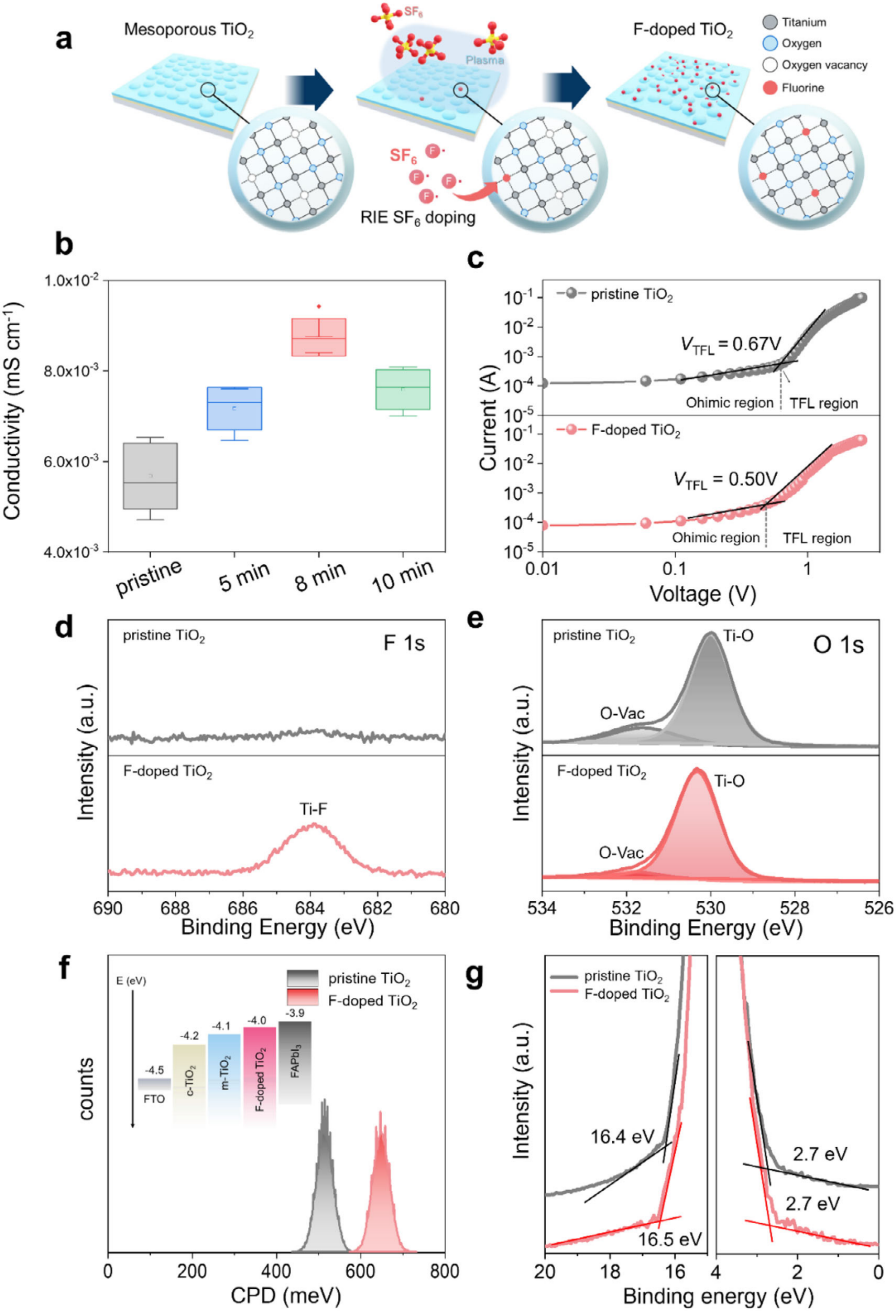
(a) Schematic illustration of the SF_6_ RIE plasma F‐doping process for TiO_2_. (b) Electrical conductivity of pristine and F‐doped TiO_2_ films at different treatment times. (c) Current–voltage curves of an electron‐only device based on pristine and F‐doped TiO_2_. XPS spectra of (d) F 1s and (e) O 1s for the pristine and F‐doped TiO_2_ films. (f) CPD distributions (inset: energy‐level diagram) [5] and (g) UPS spectra of pristine and F‐doped TiO_2._

In situ SF_6_ RIE plasma treatment was employed to incorporate F into the m‐TiO_2_ scaffold. As illustrated schematically in Figure 1a, SF_6_ plasma generates highly reactive F radicals that interact directly with the m‐TiO_2_ surface. The detailed plasma‐processing parameters are provided in the Experimental Section. To determine the optimal fluorination conditions, the electrical conductivity of m‐TiO_2_ was measured as a function of plasma exposure time. As shown in Figure 1b, all F‐doped TiO_2_ films exhibit higher electrical conductivity than the untreated films, with an 8 min treatment yielding the highest value of 9.42 × 10^−3^ S cm^−1^. The reduced standard deviation across multiple samples (Figure S1 and Table S1) further indicates improved film uniformity after fluorination. This conductivity enhancement is attributed to the passivation of oxygen‐vacancy‐related trap states, which reduces electron scattering and promotes more efficient charge‐transport within the m‐TiO_2_ layer. Top‐view and cross‐sectional scanning electron microscopy (SEM) images (Figures S2 and S3) reveal that the SF_6_ RIE plasma process does not induce structural damage to the m‐TiO_2_ scaffold. In addition, elemental mapping via SEM‐energy‐dispersive X‐ray Spectroscopy (EDS) confirms the presence of F within the m‐TiO_2_ layer after plasma treatment (Figure S4). Atomic force microscopy (AFM) images show a reduction in the root‐mean‐square (RMS) roughness from 16.79 to 14.55 nm, indicating improved surface uniformity after fluorination (Figure S5) [24]. These results suggest that the plasma‐induced fluorination effectively enhances surface uniformity of the m‐TiO_2_ without structural damage. Moreover, the water contact angle increased from 22.32° to 38.97° after SF_6_ plasma treatment (Figure S6), indicating the formation of a more hydrophobic, F‐terminated surface. This increased hydrophobicity is expected to hinder moisture penetration into the TiO_2_ framework and improve environmental stability.

Subsequently, to quantitatively evaluate the trap passivation effect, electron‐only devices (FTO/c‐TiO_2_/m‐TiO_2_/Au) were fabricated, and trap‐filled limit voltage (*V*_TFL_) was evaluated (Figure 1c). The F‐doped TiO_2_ exhibited a reduced *V*_TFL_ of 0.50 V compared to 0.67 V for the pristine TiO_2_ film, confirming a lower trap density (*n*_t_ for F‐doped and pristine TiO_2_ are 1.35 × 10^17^ and 1.81 × 10^17^ cm^−3^, respectively). These results demonstrate that F‐doping effectively reduces deep trap states, which can suppress trap‐assisted interfacial recombination [9]. X‐ray photoelectron spectroscopy (XPS) measurement was employed to investigate the chemical bonding states in the F‐doped TiO_2_ films. A distinct F 1s peak at 683.9 eV appears only in the fluorinated films (Figure 1d), corresponding to the formation of Ti─F bonds [25] and confirming successful F incorporation.

The O 1s spectrum (Figure 1e) shows a slight upward shift of the lattice‐oxygen peak, reflecting reduced electron density around oxygen atoms due to the strong electronegativity of incorporated F [26]. In addition, the quantitative analysis of oxygen vacancies revealed that the relative fraction of oxygen vacancies in TiO_2_ is markedly reduced from 17.4% to 7.6% after fluorination [27]. This pronounced decrease indicates that F species preferentially occupy oxygen vacancy sites to form Ti─F bonds, thereby effectively passivating oxygen vacancies and suppressing oxygen‐vacancy‐related Ti^3+^ defect states in TiO_2_. Furthermore, an increase in the contact potential difference (CPD) from 514.2 to 646.6 meV measured by Kelvin probe force microscopy (KPFM, Figure 1f), together with an upshift of the Fermi level observed by UV photoelectron spectroscopy (Figure 1g), consistently indicates effective n‐type doping of TiO_2_. These electronic structure changes are attributed to Ti─F coordination, which simultaneously eliminates detrimental deep defect states associated with oxygen vacancies and induces shallow donor‐like states near the conduction band, thereby shifting the Fermi level toward the conduction band [28, 29, 30]. An energy‐level diagram derived from the UPS data is presented in the inset of Figure 1f. Collectively, these characterizations demonstrate that the SF_6_ RIE plasma fluorination process effectively incorporates F into the m‐TiO_2_ scaffold, yielding a more conductive, defect‐passivated, and electronically favorable ETL, which is beneficial for efficient electron extraction at the TiO_2_/perovskite interface.

### 2.2 Properties of Perovskite Films on TiO_2_

**FIGURE 2:**
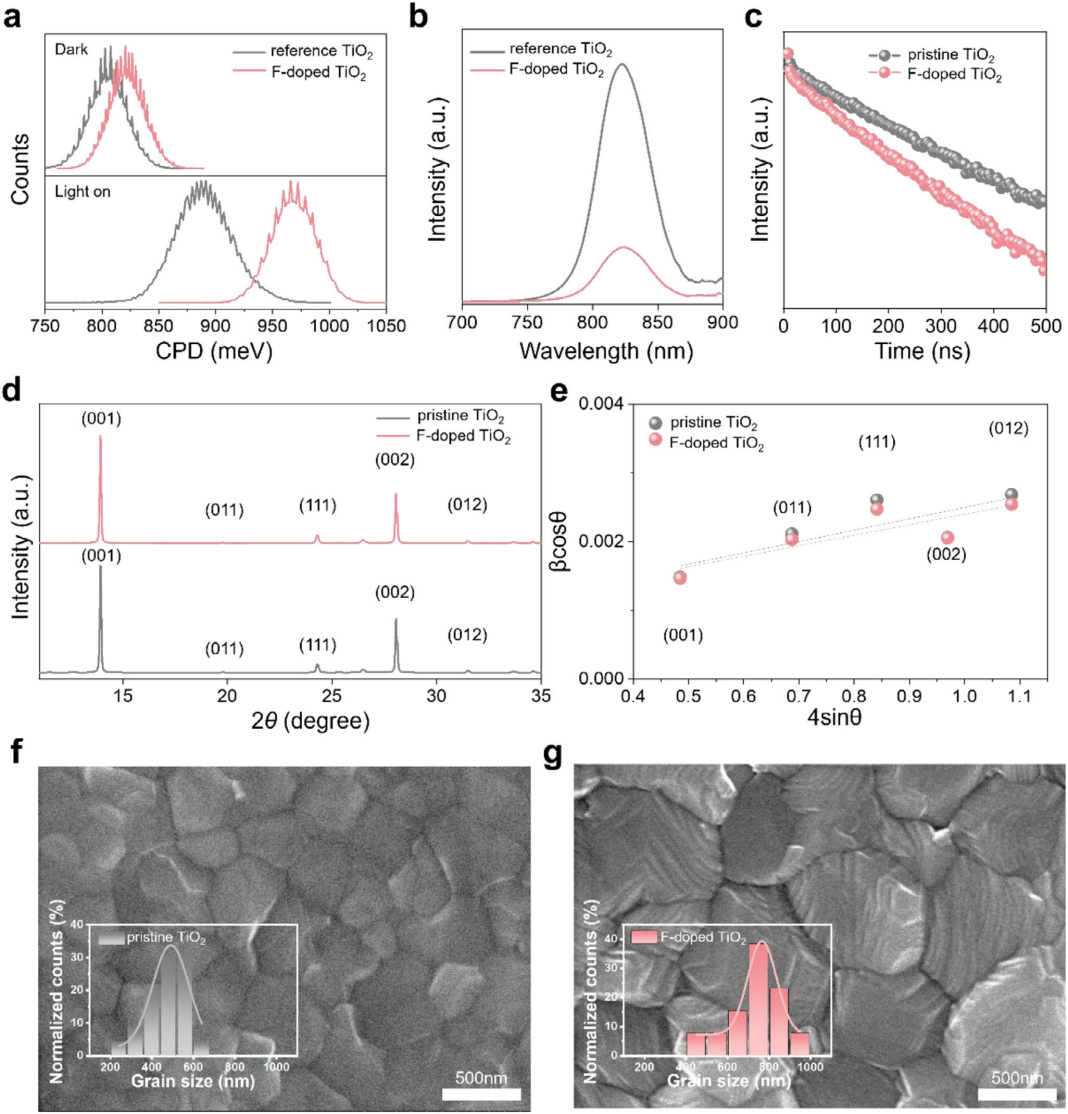
(a) CPD distribution of FAPbI_3_ films deposited on pristine and F‐doped TiO_2_ under dark and illuminated conditions. (b) Steady‐state PL spectra, (c) TRPL spectra, (d) XRD patterns, and (e) W–H plot of FAPbI_3_ films on pristine TiO_2_ and F‐doped TiO_2_. Top‐view SEM images of FAPbI_3_ films on (f) pristine TiO_2_ and (g) F‐doped TiO_2_ (inset: size distribution histograms for perovskite grains).

Subsequently, we examined the influence of F incorporation on the optoelectronic properties of the FAPbI_3_ perovskite films on TiO_2_. To probe charge carrier, KPFM measurements were performed under dark and illuminated conditions for perovskite films on pristine and F‐doped TiO_2_. As shown in Figure 2a, the film on F‐doped TiO_2_ exhibited substantial surface photovoltage (SPV = CPD_light_ − CPD_dark_) to 150 meV compared to that on pristine TiO_2_ (83 meV). This enhancement indicates improved photoinduced charge separation and reduced interfacial recombination [31, 32].

Steady‐state photoluminescence (PL) measurements further corroborate the improved charge extraction. As shown in Figure 2b, the PL intensity of the FAPbI_3_ film on the F‐doped TiO_2_ is greatly reduced compared to the film on pristine TiO_2_ due to enhanced interfacial energy alignment and faster electron extraction efficiency [27]. Time‐resolved PL (TRPL) measurements (Figure 2c; Table S2) show a decrease in the average PL lifetime from 199.9 ns on the pristine TiO_2_ to 138.1 ns on the F‐doped TiO_2_. The accelerated PL decay also reflects improved carrier extraction at the F‐doped TiO_2_/perovskite interface and reduced trap‐assisted recombination pathways [33].

The structural properties of the perovskite films were then examined by X‐ray diffraction (XRD) measurements (Figure 2d). Both films exhibit the characteristic α‐phase FAPbI_3_ reflections, including the dominant (001) peak at 14.1°, with no detectable PbI_2_ signals, confirming high‐quality perovskite formation [34]. The (001) peak of the perovskite on the F‐doped TiO_2_ shows a higher intensity with reduced full width at half maximum (FWHM), indicating enhanced crystallinity. This improvement is attributed to the improved electronic quality of the underlying F‐doped TiO_2_, which plays a critical role in regulating perovskite nucleation and crystal growth at the TiO_2_ surface (Figure S7) [35, 36]. Williamson–Hall (W–H) analysis (Figure 2e) for the FAPbI_3_ films deposited on TiO_2_ revealed that the microstrain (*ε*) decreases from 0.00165 (pristine TiO_2_) to 0.00153 (F‐doped TiO_2_), suggesting reduced lattice distortion during crystallization [37, 38]. In addition, the decreased intercept of the W–H plot indicates an increased crystallite size, evidencing improved lattice coherence within the perovskite film. Consequently, fluorination of TiO_2_ improves crystallinity of the perovskite layer while alleviating defect‐induced interfacial stress, thereby contributing to enhanced structural stability of the perovskite [39, 40].

Top‐view SEM images (Figure 2f,g) further corroborate the crystallization behavior. The perovskite film grown on F‐doped TiO_2_ exhibits larger and more uniform grains, with the average grain size increasing from ∼490 nm (on pristine TiO_2_) to ∼750 nm (on F‐doped TiO_2_). Grain‐size distribution histograms (insets of Figure 2f,g) further confirm this pronounced grain enlargement. Such enlarged grains reduce the density of grain boundaries, which is beneficial for suppressing non‐radiative recombination and facilitating charge‐transport in the perovskite layer [41, 42].

These results demonstrate that fluorination of the TiO_2_ not only passivates interfacial traps but also modulates perovskite nucleation and growth, leading to reduced *ε*, improved crystallinity, and enlarged grain sizes. The concurrent improvement in interfacial energetics and bulk film quality is expected to directly contribute to the enhanced photovoltaic performance of PSCs employing F‐doped TiO_2_ ETL [43, 44].

### 2.3 Photovoltaic Performances

**FIGURE 3:**
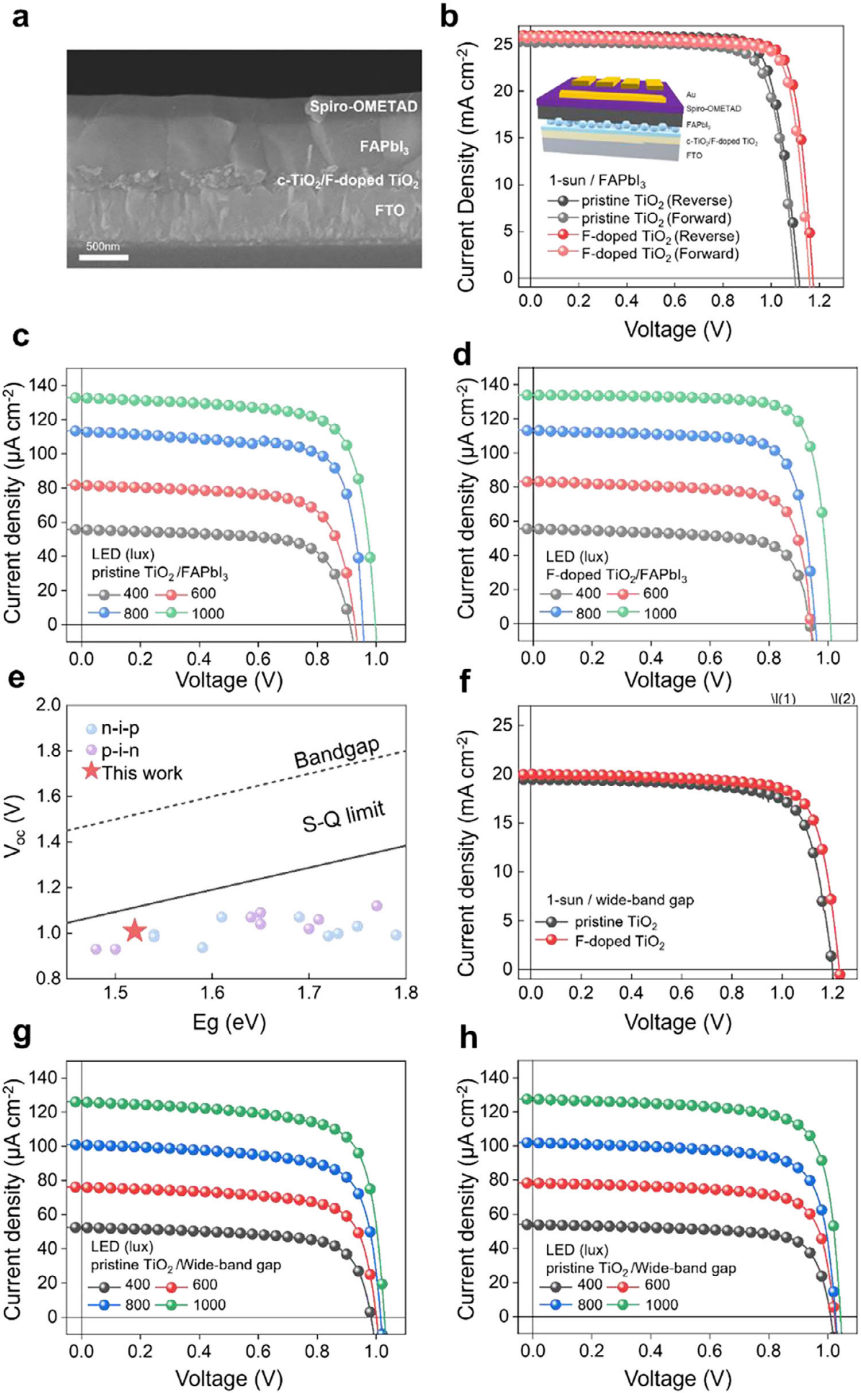
(a) Cross‐sectional SEM image of the PSC (FTO/c‐TiO_2_/m‐TiO_2_/FAPbI_3_/Spiro‐OMeTAD/Au). (b) *J–V* characteristics of PSCs employing the pristine TiO_2_ and the F‐doped TiO_2_ under one‐sun illumination. *J*–*V* curves of PSCs incorporating (c) the pristine TiO_2_ and (d) the F‐doped TiO_2_ under indoor LED illumination with 400, 600, 800, and 1000‐lux. (e) Comparison of *V*_oc_ values from this work with previously reported indoor‐light PSCs with various bandgaps, plotted with the Shockley–Queisser *V*_oc_ limit (solid line) and the optical bandgaps (dashed line). (f) *J–V* curves of wide‐bandgap PSCs on the pristine TiO_2_ and the F‐doped TiO_2_ under one‐sun illumination. *J*–*V* characteristics of wide‐bandgap PSCs fabricated on (g) the pristine TiO_2_ and (h) the F‐doped TiO_2_ under 400–1000‐lux LED illumination.

To assess the impact of F‐doping of TiO_2_ on photovoltaic behavior, we incorporated pristine and F‐doped TiO_2_ as ETLs in PSCs with the structure of FTO/c‐TiO_2_/m‐TiO_2_/FAPbI_3_/Spiro‐OMeTAD/Au (Figure 3a). PSCs employing the F‐doped TiO_2_ exhibited enhanced *V*_oc_ and FF of 1.18 and 0.82 V, compared to those based on pristine TiO_2_, 1.12 V and 0.80 V, respectively, due to suppressed trap‐assisted recombination, resulting in an increased PCE from 23.23% to 25.13% (Figure 3b; Table S3) [29, 45, 46, 47, 48, 49]. The external quantum efficiency (EQE) spectra of both PSCs yield integrated *J*_sc_ values consistent with the corresponding *J*–*V* measurements within 5% (Figure S8). Stabilized power output (SPO) measurements under continuous one‐sun illumination reveal improved operational stability for PSCs incorporating the F‐doped TiO_2_ compared to those based on the pristine TiO_2_ (Figure S9), likely due to the stabilized crystal structure of FAPbI_3_.

The performance enhancement was much markedly more pronounced under low‐intensity light illumination, where the photogenerated carrier density is extremely reduced and interfacial trap‐assisted recombination plays a more dominant role [50]. Under such conditions, F‐doping induces a substantially larger performance gain. Specifically, under 1000‐lux LED illumination, the PCE increases from 32.87% to 36.19%, which is a higher degree of increase than that observed under one‐sun illumination (Figures 3c,d). The pronounced efficiency enhancement under low‐intensity illumination implies a reduced interfacial trap density at the m‐TiO_2_/perovskite interface after fluorination owing to the effective passivation of oxygen‐vacancy‐related trap states. This is further supported by the concurrent increases in both *V*_oc_ and FF (Table S4), which are consistent with suppressed trap‐assisted recombination [51]. This reduced recombination losses also enabled a lower *V*_oc_ deficit ($V_{oc}^{SQ}-V_{oc}$) under indoor illumination compared to previously reported PSCs (Figure 3e; Table S5), underscoring the particular suitability of fluorinated TiO_2_‐based PSCs for indoor photovoltaic applications, where minimizing non‐radiative recombination is critical for maintaining high efficiency under low‐light conditions.

To confirm the broad applicability of the F‐doped TiO_2_, PSCs based on a wide‐bandgap (1.71 eV) FA_0.79_MA_0.06_Cs_0.15_Pb(I_0.7_Br_0.3_)_3_ composition were fabricated using both pristine and F‐doped TiO_2_. Under one‐sun and 1000‐lux LED illuminations, the PCE of the devices increased from 17.46% to 18.90% (Figure 3f; Table S6) and from 31.67% to 34.00% (Figure 3g,h; Table S7). These consistent improvements across perovskites with different bandgaps demonstrate that TiO_2_ fluorination is an effective and generalizable approach for enhancing PSC performance under a versatile light environment.

### 2.4 Characterizations of Recombination Dynamics

**FIGURE 4:**
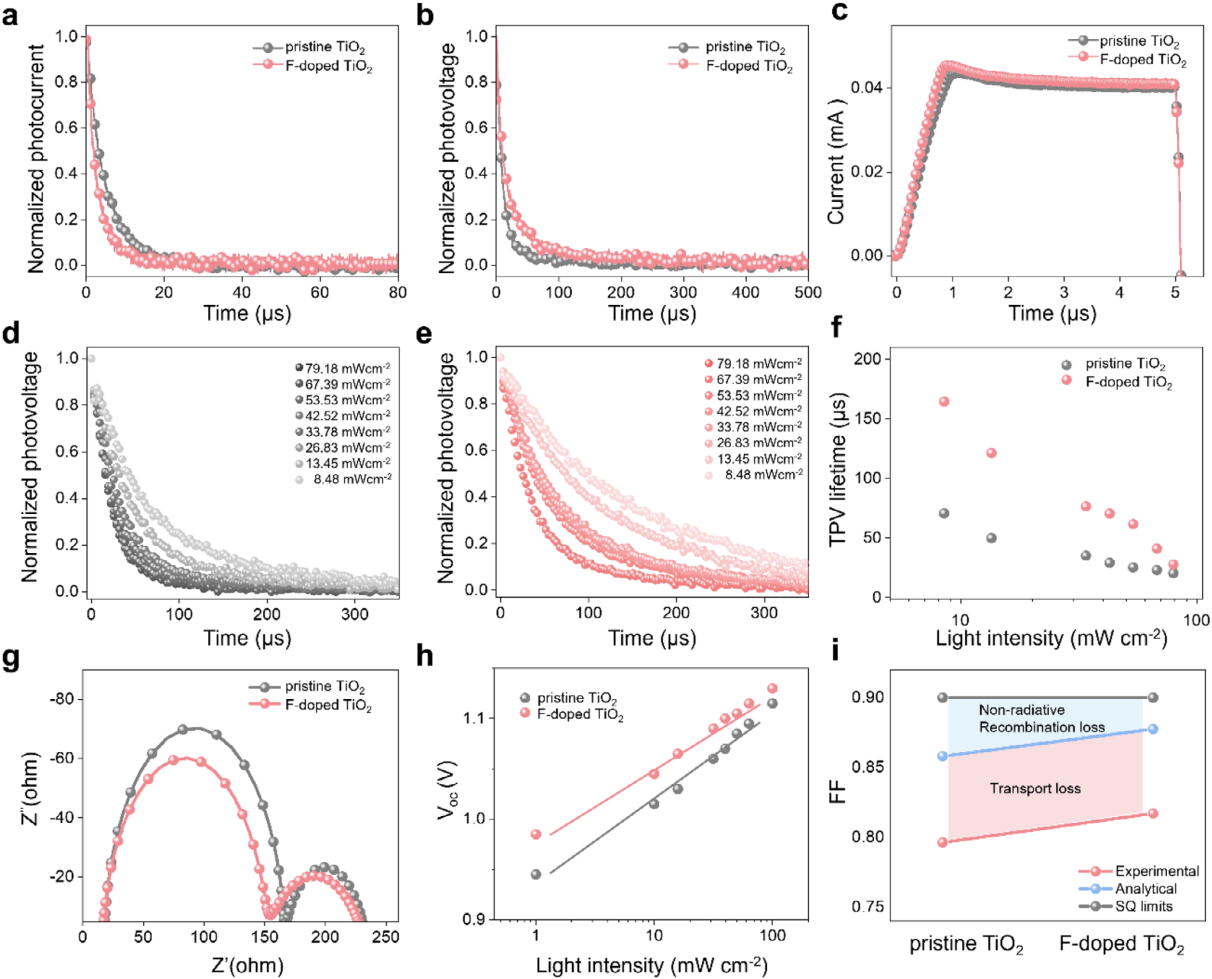
(a) TPC, (b) TPV, (c) Photo‐CELIV measurements of the PSCs with pristine and F‐doped TiO_2_. Light‐dependent TPV decay curve of PSCs based on (d) pristine and (e) F‐doped TiO_2_. (f) Light‐dependent transient photovoltage lifetime, (g) EIS Nyquist plots, and (h) *V*_oc_ changes as a function of light intensity, and (i) FF analysis of PSCs employing pristine and F‐doped TiO_2._

To elucidate the charge carrier dynamics underlying the performance enhancement, transient photocurrent (TPC), transient photovoltage (TPV), and photo‐CELIV measurements were conducted. In the TPC measurements (Figure 4a), the carrier extraction time decreases from 5.24 to 2.96 *µs* upon employing F‐doped TiO_2_, indicating faster carrier extraction [52]. TPV results (Figure 4b) reveal an increase in the carrier lifetime from 11.21 to 22.88 *µs*, confirming the suppression of trap‐assisted recombination [53]. Photo‐CELIV analysis further shows that the carrier mobility increases from 1.13 × 10^−3^ to 1.66 × 10^−3^ cm^2^ V^−1^ s^−1^ after fluorination (Figure 4c), consistent with the improved electrical conductivity of the F‐doped TiO_2_ (Figure 1a) [54]. Furthermore, electron mobility was examined by SCLC measurements using electron‐only devices with the configuration FTO/TiO_2_/perovskite/C_60_/BCP/Ag (Figure S10). Improved electron mobility in the device using F‐doped TiO_2_ (8.30 × 10^−3^ cm^2^ V^−1^ s^−1^), compared to the device using pristine TiO_2_ (6.42 × 10^−3^ cm^2^ V^−1^ s^−1^), supports enhanced electron transport capability through the F‐doping process.

To further investigate the light‐intensity dependence of recombination, TPV decay curves were recorded under a range of incident intensities (Figure 4d,e). As summarized in Figure 4f, the lifetime enhancement, i.e., the difference in carrier lifetime between PSCs using pristine and F‐doped TiO_2_, becomes particularly pronounced at low light intensities. This behavior highlights the increasingly dominant role of interfacial trap‐assisted recombination under low‐carrier‐density conditions and underscores the effectiveness of fluorination‐induced trap passivation in this regime. Consistently, this trend correlates well with the device characteristics, in which the relative PCE improvement achieved by fluorination is more pronounced under indoor illumination than under one‐sun conditions. Collectively, these results demonstrate that fluorination effectively suppresses trap‐assisted recombination even under low‐carrier‐density environments [55].

Electrochemical impedance spectroscopy (EIS) was employed to further probe interfacial charge‐transport and recombination characteristics. As shown in Figure 4g, PSCs incorporating the F‐doped TiO_2_ exhibit a lower charge‐transfer resistance (R_ct_) of 134.9 Ω than that using pristine TiO_2_ (148.2 Ω), indicating facilitated charge‐transfer at the TiO_2_/perovskite interface. Concurrently, the recombination resistance (R_rec_) increases from 66.71 to 80.08 Ω upon fluorination, confirming the effective suppression of interfacial recombination processes [56]. Consistently, Mott–Schottky analysis reveals an increase in the built‐in potential (V_bi_) from 1.07 to 1.13 V (Figure S11), implying reduced recombination‐induced voltage losses and an enhanced internal electric field at the interface, which is favorable for charge separation and extraction [57].

To further identify the dominant recombination mechanisms, the light‐intensity dependence of *V*_oc_ ​and *J*_sc_ was analyzed (Figure 4h). The slope of *V*_oc_ as a function of light intensity (ideality factor, *n*) decreased from 1.43 to 1.24 after fluorination, indicating a substantial suppression of trap‐assisted recombination [58]. This is further supported by the reduction in *V_TFL_*, which decreased from 0.33 to 0.25 V, corresponding to a decrease in *n*_t_ from 6.84 × 10^15^ to 5.18 × 10^15^ cm^−3^ (Figure S10). These results demonstrate that fluorination effectively passivates oxygen‐vacancy‐related defects and facilitates electron extraction at the ETL/perovskite interface. On the other hand, the slopes of *J*_sc_ versus light intensity for two devices are comparable (0.98 and 0.99 for PSCs based on pristine and F‐doped TiO_2_, respectively), approaching the ideal linear behavior (Figure S12) [59]. Based on the extracted *n*, analytical FF values were calculated and compared with the theoretical transport‐loss‐free FF (Figure 4i) [60]. Notably, the gap between these two FF values is significantly reduced for PSCs incorporating F‐doped TiO_2_, providing further evidence that non‐radiative losses associated with oxygen‐vacancy‐related trap states are effectively mitigated. These results consistently demonstrate that fluorination suppresses trap‐mediated recombination, enhances charge extraction efficiency, and improves interfacial charge‐transport characteristics in TiO_2_‐based PSCs [61].

### 2.5 Long‐Term Stability

**FIGURE 5:**
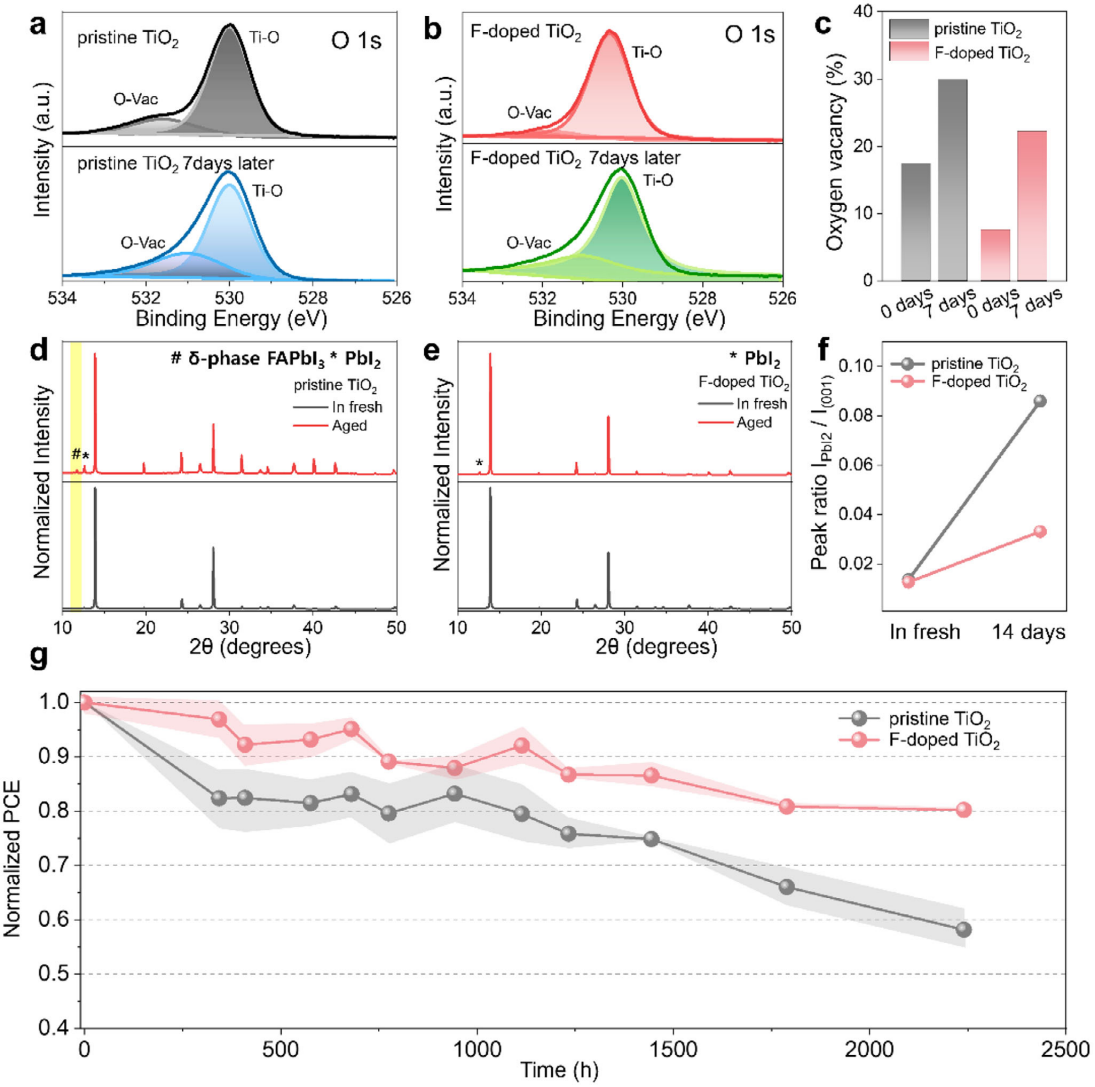
O 1s XPS spectra of (a) the pristine and (b) F‐doped TiO_2_ films, measured immediately after fabrication and after 7 days of ambient storage. (c) Oxygen‐vacancy fraction extracted from the O 1s spectra. XRD patterns of FAPbI_3_ films deposited on (d) the pristine and (e) the F‐doped TiO_2_ films, measured in the fresh state and after 14 days of ambient storage. The “#” symbol indicates the δ‐phase FAPbI_3_ reflection, and the “*” symbol indicates PbI_2_ reflection. (f) Evolution of the PbI_2_/FAPbI_3_ (001) peak‐intensity ratio in perovskite films on pristine and F‐doped TiO_2_ over 14 days. (g) Long‐term stability of un‐encapsulated FAPbI_3_ PSCs based on pristine and F‐doped TiO_2_ under ambient storage.

We evaluated the environmental stability of FAPbI_3_ PSCs based on pristine and F‐doped TiO_2_. To assess the intrinsic stability of the m‐TiO_2_, O 1s XPS spectra were collected from pristine and F‐doped TiO_2_ films stored in ambient air for 7 days (Figure 5a,b). As summarized in Figure 5c, pristine TiO_2_ exhibits an oxygen‐vacancy fraction of 17.4%, whereas F‐doped TiO_2_ shows a significantly lower value of 7.6%. After aging, the oxygen‐vacancy fraction of pristine TiO_2_ increases markedly to 29.9%, while F‐doped TiO_2_ maintains a comparatively lower oxygen‐vacancy fraction of 22.2% (Figure 5c) [62]. These results indicate that the F‐doped TiO_2_ retains a lower density of oxygen vacancies upon ambient exposure, due to the formation of stable Ti─F bonds [45] and the enhanced surface hydrophobicity imparted by fluorine incorporation [63].

We next investigated the stability of FAPbI_3_ films deposited on the two TiO_2_ after 14 days of ambient storage. XRD patterns of the perovskite films on the pristine TiO_2_ show the emergence of characteristic δ‐phase FAPbI_3_ and PbI_2_ reflections at 11.8° and 12.6°, respectively, indicating pronounced phase degradation (Figure 5d) [64, 65, 66]. In contrast, the perovskite film on the F‐doped TiO_2_ retains the α‐phase diffraction features with substantially suppressed PbI_2_ formation (Figure 5e). Quantitative peak‐area analysis further reveals that the PbI_2_/FAPbI_3_ ratio increases to 8.59% on the pristine TiO_2_, whereas it remains significantly lower at 3.32% on the F‐doped TiO_2_ (Figure 5f). This marked difference is attributed to the reduced moisture susceptibility and alleviated interfacial lattice strain afforded by fluorination, which collectively suppresses the α‐to‐δ phase transition during ambient exposure.

Finally, the long‐term operational stability of un‐encapsulated PSCs was evaluated under ambient conditions (25°C and 40% relative humidity). Devices incorporating the pristine TiO_2_ degraded rapidly, retaining less than 80% of their initial PCE after 1110 h storage. In contrast, PSCs employing the F‐doped TiO_2_ preserve more than 80% of their initial PCE even after 2240 h of storage, demonstrating substantially enhanced long‐term stability (Figure 5g). These results confirm that TiO_2_ fluorination not only improves the chemical stability of the TiO_2_ but also enhances the structural stability of the overlying perovskite layer, thereby effectively mitigating long‐term performance degradation under ambient conditions.

## 3 Conclusion

In this study, we demonstrated that fluorination of TiO_2_ via an SF_6_ RIE plasma process simultaneously enhances both photovoltaic properties and environmental stability of PSCs. XPS and trap density analyses confirmed that fluorine incorporation effectively suppresses numerous trap states in TiO_2_, while KPFM and UPS measurements revealed a shift of the Fermi level after fluorination. In addition, F‐doped TiO_2_ also promoted the formation of FAPbI_3_ films with larger grains, reduced microstrain, and improved crystallinity. These interfacial and structural improvements collectively lead to more efficient charge extraction and suppressed trap‐assisted recombination, as evidenced by enhanced SPV, reduced PL intensity, and reduced PL lifetimes. Consequently, PSCs employing the F‐doped TiO_2_ achieved a PCE of 25.13% under one‐sun illumination, accompanied by increases in both *V*_oc_ and FF, compared to pristine TiO_2_ devices. Notably, under 1000‐lux LED illumination, the PCE was further improved to 36.19%, highlighting the pronounced reduction of defect‐mediated recombination losses under a low‐intensity light environment. The effectiveness of this fluorination strategy was further validated for wide‐bandgap perovskite compositions, demonstrating its general applicability. Finally, F‐doped TiO_2_ effectively suppressed the formation of δ‐phase FAPbI_3_ and PbI_2_ during ambient aging, enabling un‐encapsulated devices to retain over 80% of their initial efficiency even after 2000 h of storage. Overall, SF_6_ RIE plasma fluorination offers a direct, controllable, and scalable approach for defect passivation and electronic‐structure modulation in TiO_2_, providing an effective route toward simultaneously achieving high efficiency and long‐term stability in PSCs.

## 4 Experimental Section

### 4.1 Materials

Formamidinium iodide (FAI), formamidinium bromide (FABr), and methylammonium bromide (MABr) were purchased from Greatcell Solar. Titanium diisopropoxide bis(acetylacetonate), methylammonium chloride (MACl, 98%), octylammonium iodide (OAI), cesium iodide (CsI), 4‐tert‐butylpyridine (tBP), lithium bis(trifluoromethane)sulfonyl imide lithium salt (Li‐TFSI), FK 209 Co(III) TFSI salt (FK209), N, N‐dimethylformamide (DMF, 99.8%), chlorobenzene (CB, 99.8%), dimethyl sulfoxide (DMSO, 99.9%), diethyl ether (DEE, 99.7%), isopropanol (IPA), acetonitrile (ACN, 99.8%), and 2‐methoxyethanol (99.8%) were purchased from Sigma–Aldrich. TiO_2_ paste (SC‐HT040) was obtained from ShareChem. Lead iodide (PbI_2_, 99.99%), and lead bromide (PbBr_2_, 99.99%) were purchased from TCI. All chemicals were used as received without further purification unless otherwise specified.

### 4.2 FAPbI_3_ Powder Synthesis

2.378 g of FAI and 6.072 g of PbI_2_ were dissolved in 20 mL of 2‐methoxyethanol to form a concentrated precursor solution. The solution was stirred at 40°C for 30 min to ensure complete dissolution. The resulting yellow solution was then heated at 100°C under stirring, followed by recrystallization using a retrograde method. The obtained powder was filtered and baked at 150°C for 30 min. Finally, the synthesized FAPbI_3_ powder was dried overnight in a vacuum oven at 60°C before use.

### 4.3 Device Fabrication

Fluorine‐doped tin oxide (FTO, Asahi) coated glass substrates were sequentially cleaned in an ultrasonic bath with detergents, deionized water, acetone, and isopropanol, each for 15 min. A compact TiO_2_ (c‐TiO_2_) layer was deposited onto the cleaned FTO substrates via spray pyrolysis at 450°C using a precursor solution of titanium diisopropoxide bis(acetylacetonate) in ethanol (1:10 volume ratio), sprayed for 30 min (temperature: 25°C and relative humidity: 30%). After cooling to room‐temperature, a mesoporous TiO_2_ (m‐TiO_2_) layer was formed by spin‐coating a TiO_2_ paste (275 mg in 1.5 mL ethanol/terpineol, 78:22 volume ratio) at 3000 rpm for 30 s, followed by annealing at 500°C for 1 h in ambient air.

The substrates with the c‐ TiO_2_/m‐TiO_2_ layers were transferred to a reactive ion etching (RIE) chamber for F‐doping. The SF_6_ RIE plasma treatment was conducted for 8 min under the following conditions: a radio frequency (RF) power of 15 W, a gas flow rate of 10 sccm, and a working chamber pressure of 0.3 Torr. The following fabrication processes were performed in a nitrogen‐filled glovebox. The FAPbI_3_ perovskite precursor solution was prepared by dissolving 1139 mg of FAPbI_3_ and 42 mg of MACl in a mixed solvent of DMF and DMSO (4:1 v/v). FAPbI_3_ solution was spin‐coated onto the TiO_2_ layer in a two‐step process at 500 rpm for 5 s and then at 6000 rpm for 50 s. During the second step, 1 mL of DEE was dropped 45 s before the end of the program. The resulting films were immediately annealed on a hotplate at 150°C for 10 min.

After the substrates cooled to room‐temperature, the Spiro‐OMeTAD solution (90 mg mL^−1^ in CB) with the Li‐TFSI (23.66 µL of 520 mg mL^−1^ in ACN), tBP (35.5 µL), and FK209 (9.08 µL of 360 mg mL^−1^ in ACN) was deposited by spin‐coating at 3000 rpm for 30 s. Finally, a 100 nm thick gold (Au) top electrode was deposited by thermal evaporation under a high vacuum condition (<1 × 10^−6^ Torr). The active area of the device was defined as 0.105 cm^2^ using a metal shadow mask.

### 4.4 FA_0.79_MA_0.06_Cs_0.15_Pb(I_0.7_Br_0.3_)_3_ Perovskite Fabrication

FA_0.79_MA_0.06_Cs_0.15_Pb(I_0.7_Br_0.3_)_3_ perovskite films were fabricated using a mixed halide precursor solution prepared by dissolving FAI (128 mg), PbI_2_ (446 mg), MABr (9.07 mg), PbBr_2_ (150 mg), CsI (52 mg), FABr (40.45 mg), and MACl (21 mg) in a mixed solvent of DMF/DMSO (4:1 v/v). The precursor solution was spin‐coated onto the substrates in a two‐step 1000 rpm for 10 s, 4000 rpm for 40 s. During the second step, 150 µL of CB was dropped after 20 s. The resulting films were annealed at 150°C for 10 min. All subsequent procedures were identical to those described for FAPbI_3_ fabrication.

### 4.5 Characterization

All device measurements were performed under ambient laboratory conditions (temperature: 25°C, relative humidity: 30%). The cross‐sectional and top‐view morphologies are characterized by field‐emission scanning electron microscopy (FE‐SEM, Hitachi S‐4800). The crystallinity and phase were examined by X‐ray diffraction (XRD, SmartLab (9 kW), Rigaku) using a Cu Kα radiation source (λ = 1.54 Å). X‐ray photoelectron spectroscopy (XPS) was analyzed by Thermo Fisher Scientific K‐Alpha+. Steady‐state photoluminescence (PL) and time‐resolved photoluminescence (TRPL) spectra were conducted by Edinburgh Instruments FS5. Atomic force microscopy (AFM) and Kelvin probe force microscopy (KPFM) were performed using a Park Systems XE‐7 instrument in a nitrogen‐filled glovebox. UV photoelectron spectroscopy was performed by Thermo Fisher Scientific. Space‐charge‐limited current (SCLC) measurements of electron‐only devices (FTO/TiO_2_/Au, FTO/TiO_2_/Perovskite/C_60_/BCP/Ag) were performed in the dark using a Keithley 4200‐SCS with a scan step of 0.01 V. The conductivity of the TiO_2_ films was measured using a Keithley 4200‐SCS. The current density–voltage (*J*–*V*) characteristics of the perovskite solar cells were measured using a potentiostat (IVIUM, Compactstat) under simulated AM 1.5G illumination (100 mW/cm^2^) from a solar simulator (Oriel LCS‐100, Newport). The light intensity was calibrated using KG‐5 reference silicon solar cell. The step voltage was 5 mV, and the measurement speed was 400 mV s^−1^. For indoor photovoltaic performance evaluation, a dedicated LED illumination system (Mcscience, K3000) was used, and the light intensity was calibrated to 400, 600, 800, and 1000‐lux using a luxmeter (GL Optic, Spectrolux). EQE spectra were measured by using an incident‐photon‐to‐current conversion efficiency setup with a power source (450 W Xenon lamp, Newport) and a monochromator, which was calibrated using a reference Silicon photovoltaic cell. Transient photocurrent (TPC) and transient photovoltage (TPV) measurements were conducted by using a semiconductor parameter system (Mcscience, T4000) with 85 mW cm^−2^ LED illumination. The light intensity of TPV excitation was controlled by using a neutral density (ND) filter. EIS (near *V*_oc_ bias voltage, 10 Hz^−1^ MHZ under one‐sun conditions) and Mott–Schottky (The tested frequency was 10 000 Hz) were carried out using a potentiostat (Compactstat, IVIUM).

## Conflicts of Interest

The authors declare no conflicts of interest.

## Supporting information




**Supporting Information**: advs75021‐sup‐0001‐SuppMat.docx

## Data Availability

The data that support the findings of this study are available from the corresponding author upon reasonable request.
