# Supplementary material for: Fluorine‐Doping of Mesoporous TiO2 Enables Efficient Defect Passivation for High‐Efficiency and Stable Perovskite Solar Cells
Source: Adv Sci (Weinh). 2026 Apr 2;13(33):e24360. doi: 10.1002/advs.202524360 (PMC13271601; doi:10.1002/advs.202524360)
Supplement: Supplementary file 1 — Supporting Information: advs75021‐sup‐0001‐SuppMat.docx [file ADVS-13-e24360-s001.docx]

Supporting Information

Fluorine-Doping of Mesoporous TiO_2_ Enables Efficient Defect Passivation for High-Efficiency and Stable Perovskite Solar Cells

*Hye W. Chun, Sang Yeon Lee, Sang Eun Yoon, Gyeong G. Jeon, Veera Murugan Arivunithi, So Jeong Shin, Min Jun Choi, Byeongsu Kim, Min-Ho Lee, Dohyeon Jeon, Taekyeong Kim*, Jung-Yong Lee*, Jong H. Kim**

H. W. Chun, S. E. Yoon, G. G. Jeon, V. M. Arivunithi, S. J. Shin, M. J. Choi and J. H. Kim

Department of Molecular Science and Technology, Ajou University, Suwon, 16499, Republic of Korea.

E-mail: jonghkim@ajou.ac.kr

S. Y. Lee, M-H Lee, J-Y. Lee

School of Electrical Engineering, Korea Advanced Institute of Science and Technology (KAIST), Daejeon 34141, Republic of Korea

E-mail: jungyong.lee@kaist.ac.kr

B. Kim

School of Electrical Engineering, Korea Advanced Institute of Science and Technology (KAIST), Daejeon 34141, Republic of Korea

School of Electrical and Electronic Engineering, University of Ulsan, Ulsan 44610, Republic of Korea

D. Jeon, T. Kim

Department of Physics, Hankuk University of Foreign Studies, Yongin 17035, Republic of Korea

E-mail: tkim@hufs.ac.kr

Hye W. Chun and Sang Yeon Lee contributed equally to this work.

**1. Williamson-Hall (W-H) method**

The Williamson-Hall (W-H) analysis was used to evaluate the crystallite size and microstrain of the perovskite films. In this method, the total peak broadening ($\beta_{T}$) in the XRD pattern is separated into size broadening ($\beta_{D}$) and strain broadening ($\beta_{\varepsilon}$) expressed as:

$$\beta_{T} cos \theta= \varepsilon(4 sin \theta) + K\lambda/D$$

Where $\varepsilon$ is the microstrain, D is the crystallite size, K is the shape factor (0.9), $\lambda$is the X-ray wavelength, and $\theta$ is the Bragg angle. From the linear plot of $\beta_{T} cos \theta$versus $4 sin \theta$, the slope gives the microstrain ($\varepsilon$), and y-intercept provides the crystallite size (D).^[1]^

**2. Fill factor loss deconvolution analysis**

To analyze the origin of fill factor (FF) loss, the experimentally measured FF (FF_exp_) was compared to the Shockley-Queisser (S-Q) limit (FF_SQ_). The FF loss can be divided into two main contributions: non radiative recombination loss and charge transport loss. The maximum FF (FF_max_), which accounts for nonradiative recombination but excludes charge transport losses, was calculated using the following relation

$$\mathrm{FF}_{\max}= \frac{v_{oc}-\ln\left( v_{oc}+0.72 \right)}{v_{oc}+1}, \mathrm{with} v_{oc}= \frac{qV_{OC}}{nK_{B}T}$$

Here, q is the elementary charge, K_B_ is the Boltzmann constant, T is the absolute temperature (300 K) and n is the diode ideality factor extracted from the slope of light intensity-dependent *V_oc_* plot. The nonradiative FF loss is quantified as the difference between FF_SQ_ and FF_max_, while the charge transport loss is given by the difference between FF_max_ and the experimental FF_exp_.^[2]^

_
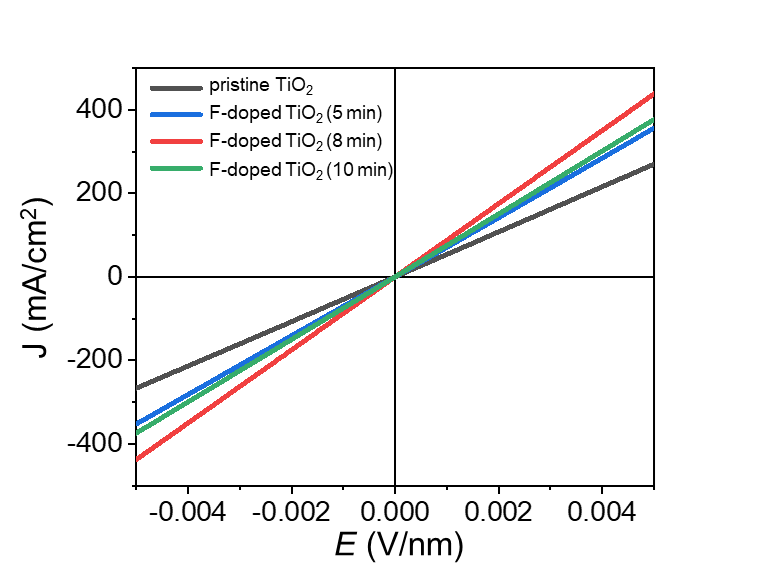
_

**Figure S1.** Current density - Electric field (J-E) curve of pristine TiO_2_ and F-doped TiO_2_ films treat for 5, 8, 10 min.


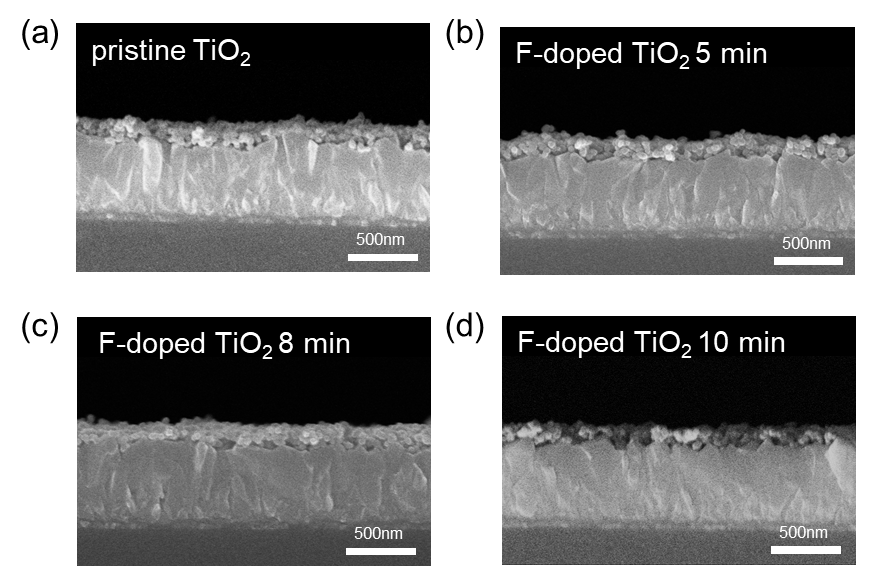


**Figure S2.** Cross sectional SEM images of (a) pristine TiO_2_ and F-doped TiO_2_ films treat for (b) 5 min, (c) 8 min, (d) 10 min.


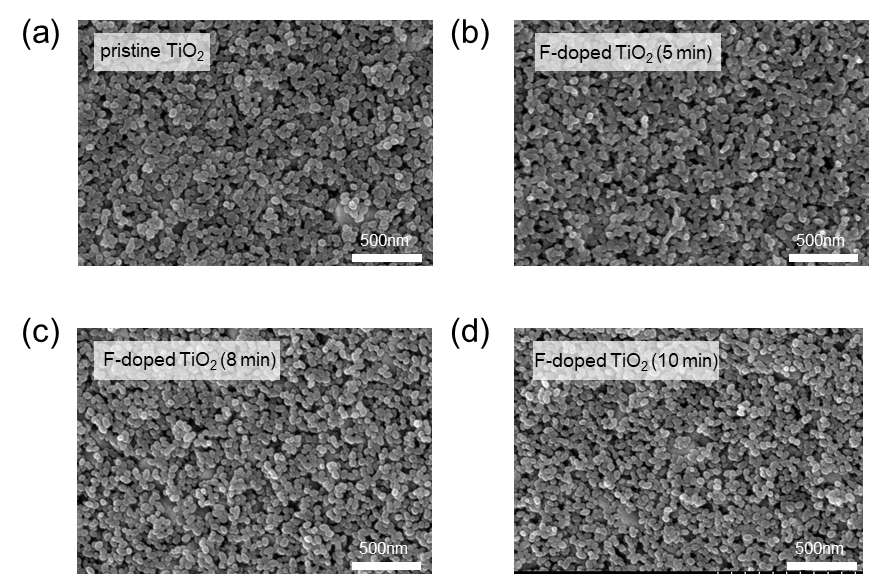


**Figure S3**. Top view SEM images of (a) pristine TiO_2_ and F-doped TiO_2_ films treats for (b) 5min, (c) 8min and (d) 10min


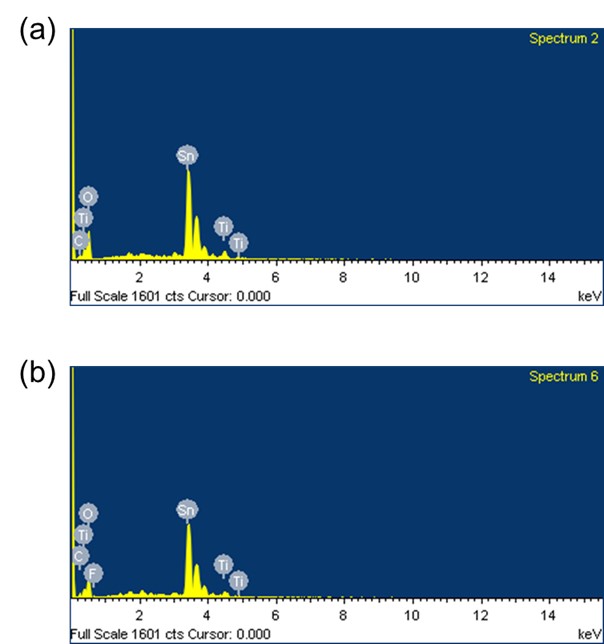


**Figure S4.** Energy dispersive spectra (EDS) of (a) pristine TiO_2_ and (b) F-doped TiO_2_ films.


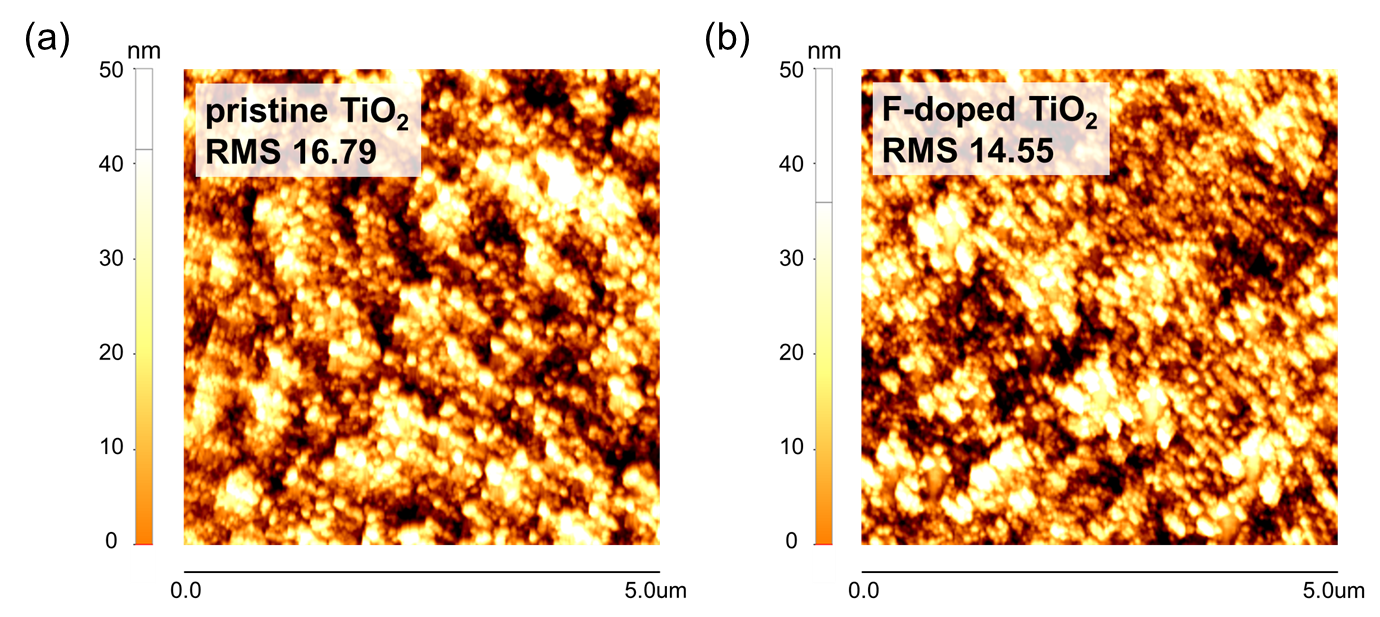


**Figure S5**. AFM images of (a) pristine TiO_2_ and (b) F-doped TiO_2_ films


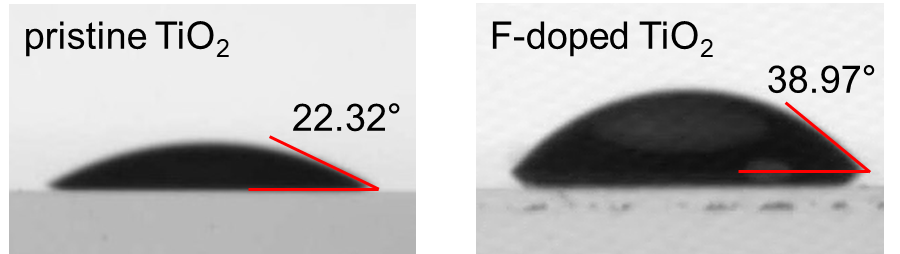


**Figure S6**. Contact angle measurements of (a) pristine TiO_2_ and (b) F-doped TiO_2_ films


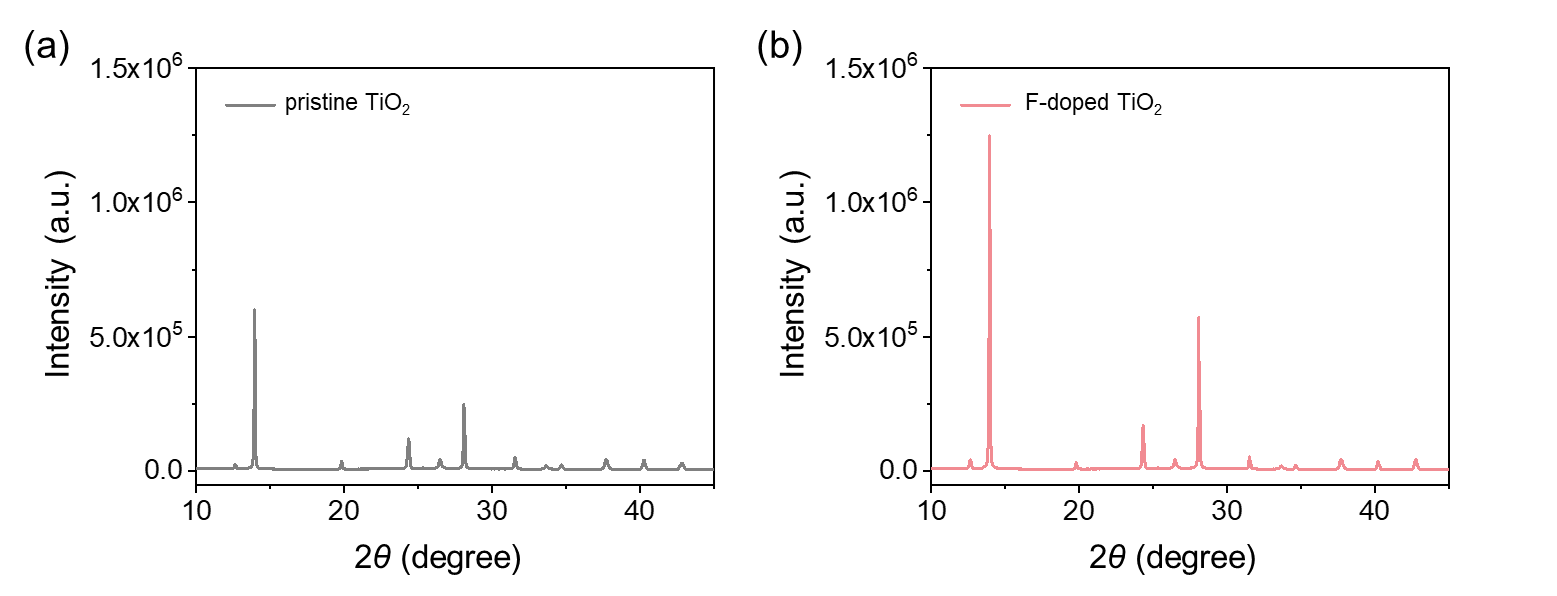


**Figure S7**. XRD spectra of (a) pristine TiO_2_ and (b) F-doped TiO_2_ films


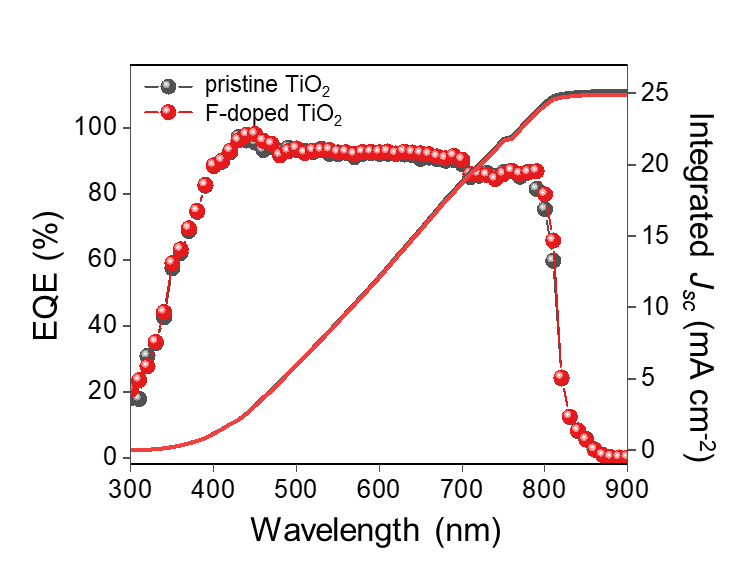


**Figure S8.** EQE spectra and corresponding integrated *J*_sc_ of PSCs based on pristine TiO_2_ (25.14 mA cm^-2^) and F-doped TiO_2_ (24.89 mA cm^-2^).


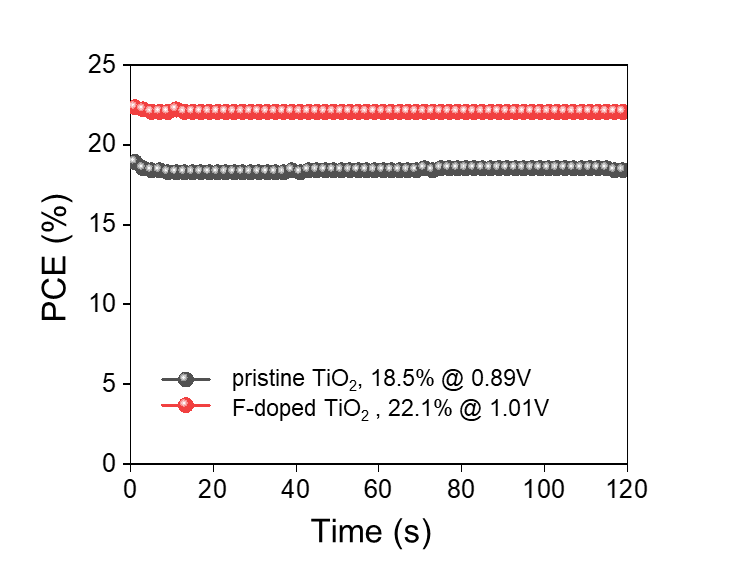


**Figure S9.** Stabilized power output (SPO) of the pristine TiO_2_ and F-doped TiO_2_ PSCs.


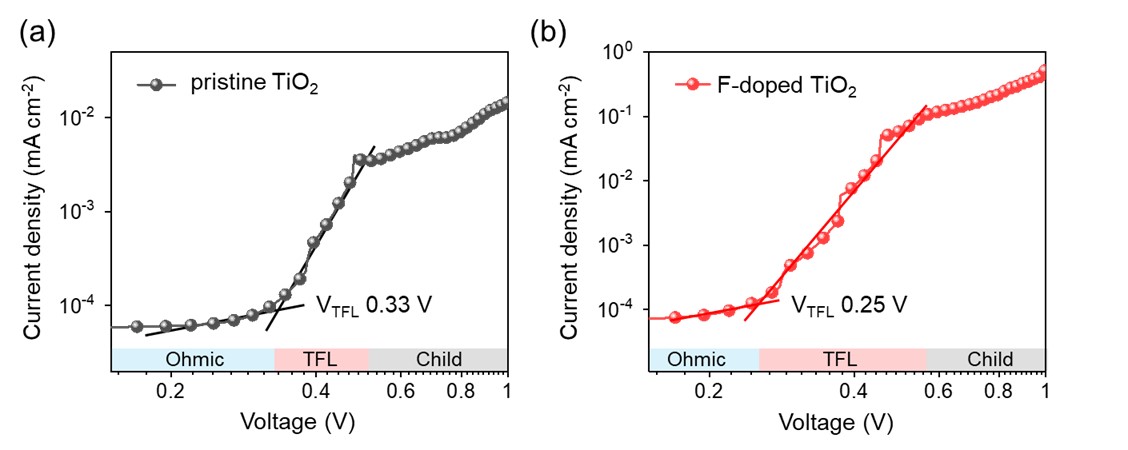


**Figure S10.**  *J*–*V* curves of electron-only devices based on pristine and F-doped TiO_2_ PSCs (FTO/TiO_2_/Perovsktie/C_60_/BCP/Ag).


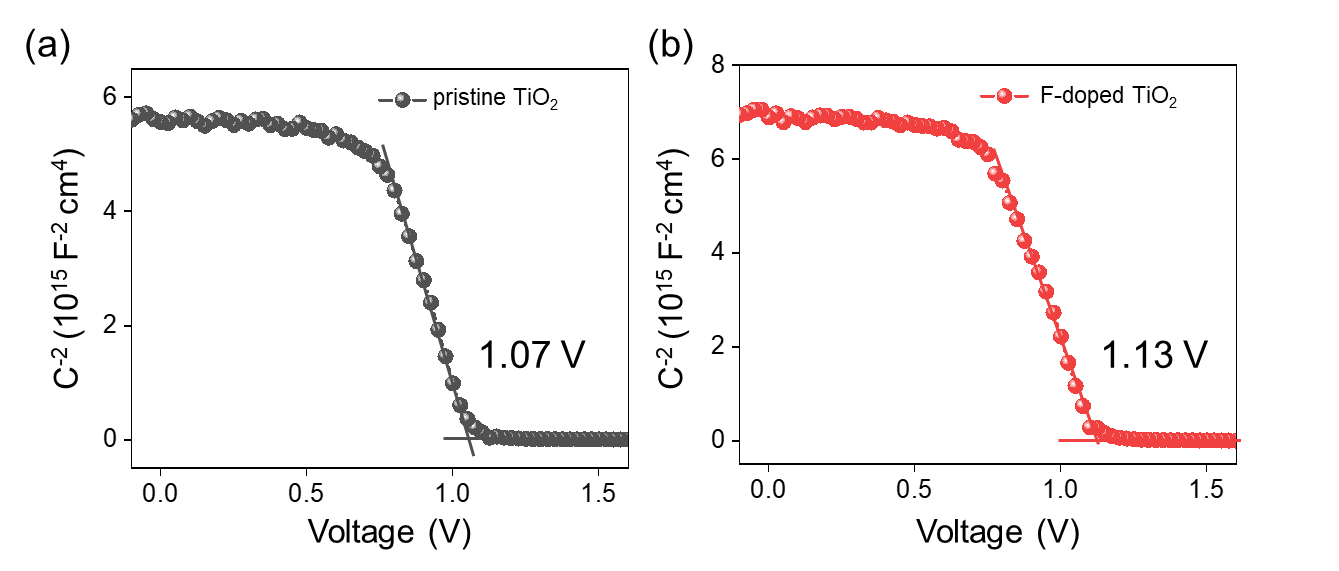


**Figure S11.** Mott-Schottky plot of (a) pristine TiO_2_ , (b)F-doped TiO_2_ PSCs.


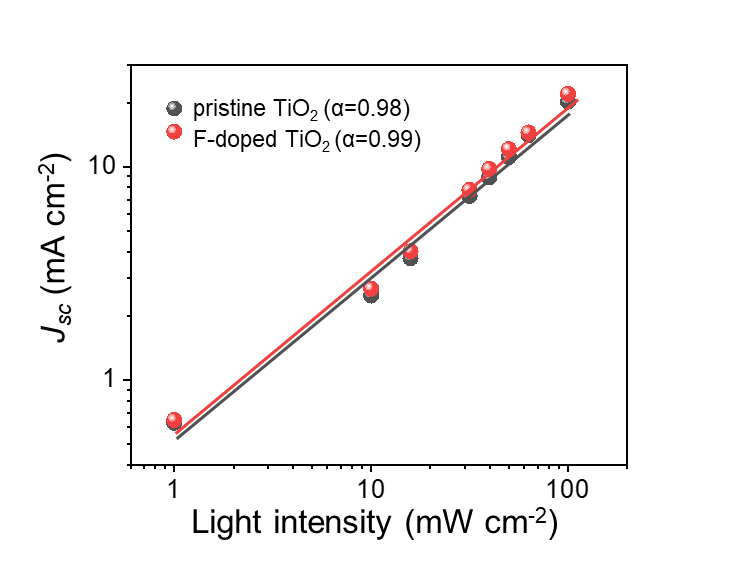
\

**Figure S12.** Light intensity dependent *J*_sc_ plot.

**Table S1.** Electrical conductivity of TiO_2_ films as a function of plasma treatment time

| Treatment time (min) ^a)^ | Average conductivity  [S cm^−1^] | Best conductivity  [S cm^−1^] | Standard deviation |
| --- | --- | --- | --- |
| 0 min  (pristine) | 5.68×10^−3^ | 6.53×10^−3^ | 7.26×10^−4^ |
| 5 min | 7.17×10^−3^ | 7.60×10^−3^ | 4.70×10^−4^ |
| 8 min | 8.74×10^−3^ | 9.43×10^−3^ | 4.17×10^−4^ |
| 10 min | 7.59×10^−3^ | 8.09×10^−3^ | 4.39×10^−4^ |

1. The average conductivity was obtained for 5 films.

**Table S2.** Detailed time-resolved PL parameters of perovskite films on pristine TiO_2_ and F-doped TiO_2_. The average lifetime was calculated from $\boldsymbol{\tau}_{\boldsymbol{ave}}$ = ($\boldsymbol{\tau}_{\boldsymbol{1}}$^2^**A_1_** + $\boldsymbol{\tau}_{\boldsymbol{2}}$^2^**A_2_**)/($\boldsymbol{\tau}_{\boldsymbol{1}}$**A_1_** + $\boldsymbol{\tau}_{\boldsymbol{2}}$**A_2_**).

| Condition | Bi-exponential fit | | | Decay amplitude ratio | | Average | |
| --- | --- | --- | --- | --- | --- | --- | --- |
|  | $\boldsymbol{\tau}_{\boldsymbol{1}}$ [ns] | $\boldsymbol{\tau}_{\boldsymbol{2}}$ [ns] | A_1_ [%] | | A_2_ [%] | | $\boldsymbol{\tau}_{\boldsymbol{ave}}$ [ns] |
| pristine TiO_2_ | 4.55 | 205.85 | 56.6 | | 43.4 | | 199.9 |
| F-doped TiO_2_ | 2.98 | 151.81 | 83.7 | | 16.3 | | 138.1 |

**Table S3**. Detailed photovoltaic properties for FAPbI_3_ PSCs under 1-sun illumination.

| Conditions ^a)^ | Scan direction | *V*_oc_  [V] | *J*_sc_  [mA cm^−2^] | FF | PCE  [%] |
| --- | --- | --- | --- | --- | --- |
| pristine TiO_2_ | FS | 1.10 | 25.24 | 0.78 | 21.66 |
|  | RS | 1.12 | 25.93 | 0.80 | 23.23 |
|  |  | (1.12) | (25.50) | (0.80) | (22.86) |
| F-doped TiO_2_ | FS | 1.16 | 25.76 | 0.81 | 24.20 |
|  | RS | 1.18 | 25.97 | 0.82 | 25.13 |
|  |  | (1.16) | (25.73) | (0.82) | (24.55) |

1. The average photovoltaic parameters of PSCs were obtained for 10 devices.

**Table S4**. Detailed photovoltaic properties for FAPbI_3_ PSCs under different light intensity LED illumination.

| Conditions | LED  [lux] | *V*_oc_  [V] | *J*_sc_  [μA cm^−2^] | FF | Power density  [μW cm^−2^] | iPCE  [%] |
| --- | --- | --- | --- | --- | --- | --- |
| pristine TiO_2_ | 1000 | 1.00 | 132.79 | 0.74 | 98.26 | 32.87 |
|  | 800 | 0.96 | 113.24 | 0.74 | 80.45 | 33.26 |
|  | 600 | 0.93 | 81.57 | 0.71 | 53.86 | 29.70 |
|  | 400 | 0.92 | 47.65 | 0.69 | 30.25 | 29.06 |
| F-doped TiO_2_ | 1000 | 1.01 | 133.88 | 0.80 | 108.18 | 36.19 |
|  | 800 | 0.96 | 113.95 | 0.77 | 84.23 | 34.82 |
|  | 600 | 0.95 | 83.34 | 0.75 | 59.38 | 32.74 |
|  | 400 | 0.94 | 55.58 | 0.72 | 37.62 | 30.98 |

**Table S5.** Summary of *V_oc_* deficit and related photovoltaic parameters for indoor PSCs under low-light (1000 lux LED) conditions.

|  | Light | lux | Perovskite composition | Structure | E_g_ [eV] | $V_{oc}^{SQ}$ | *V*_oc_  [V] | *V*_oc_  deficit | Ref. |
| --- | --- | --- | --- | --- | --- | --- | --- | --- | --- |
|  | LED | 1000 | FAPbI_3_ | nip | 1.52 | 1.11 | 1.01 | 0.01 | This work |
|  | LED | 1002 | FAPbI_3_ | nip | 1.54 | 1.13 | 0.99 | 0.14 | [3] |
|  | LED | 1000.5 | FA_0.85_MA_0.15_PbI_3_ | nip | 1.54 | 1.13 | 0.98 | 0.15 | [4] |
|  | LED | 1000 | (FAPbI_3_)_0.85_(MAPbBr_3_)_0.15_ | nip | 1.59 | 1.18 | 0.94 | 0.24 | [5] |
|  | LED | 984 | Cs_0.05_FA_0.85_MA_0.10_Pb(I_0.97_Br_0.03_)_3_ | nip | 1.61 | 1.20 | 1.07 | 0.13 | [6] |
|  | LED | 1062 | CsPbI_3_ | nip | 1.69 | 1.28 | 1.07 | 0.21 | [7] |
|  | LED | 1000 | CsPbI_3_ | nip | 1.72 | 1.31 | 0.99 | 0.32 | [8] |
|  | LED | 1000 | CsPbI_2.7_Br_0.3_ | nip | 1.73 | 1.32 | 1.00 | 0.32 | [9] |
|  | LED | 1000 | FA_0.64_MA_0.36_Pb(I_0.64_Br_0.36_)_3_ | nip | 1.75 | 1.34 | 1.03 | 0.31 | [10] |
|  | LED | 1000 | CsPbI_3_ QD | nip | 1.79 | 1.37 | 0.98 | 0.39 | [11] |
|  | LED | 1000 | Cs_0.15_FA_0.85_PbI_3_ | pin | 1.48 | 1.07 | 0.93 | 0.14 | [12] |
|  | LED | 1000 | MAPbI_3_ | pin | 1.50 | 1.09 | 0.93 | 0.16 | [13] |
|  | LED | 1000 | Cs_0.18_FA _0.82_Pb(I_0.8_Br_0.2_)_3_ | pin | 1.65 | 1.24 | 1.09 | 0.15 | [14] |
| 14 | LED | 1000 | Cs_0.18_FA _0.82_Pb(I_0.8_Br_0.2_)_3_ | pin | 1.65 | 1.24 | 1.04 | 0.20 | [15] |
| 15 | LED | 1000 | CsPbI_3_ | pin | 1.70 | 1.29 | 1.02 | 0.27 | [16] |
| 16 | LED | 1000 | Cs_0.05_FA_0.70_MA_0.25_PbI_2.25_Br_0.75_ | pin | 1.71 | 1.30 | 1.07 | 0.23 | [17] |
| 17 | LED | 1000 | CsPbI_2_Br | pin | 1.93 | 1.51 | 1.04 | 0.47 | [18] |

**Table S6.** Detailed photovoltaic properties for FA_0.79_MA_0.06_Cs_0.15_Pb(I_0.7_Br_0.3_)_3_ PSCs under 1sun illumination.

| Conditions ^a)^ |  | *V*_oc_  [V] | *J*_sc_  [mA cm^-2^] | FF | PCE  [%] |
| --- | --- | --- | --- | --- | --- |
| pristine TiO_2_ | maximum  (average) | 1.21 | 19.50 | 0.74 | 17.46 |
|  |  | (1.21) | (19.62) | (0.73) | (17.30) |
| F-doped TiO_2_ | maximum  (average) | 1.23 | 19.96 | 0.77 | 18.90 |
|  |  | (1.23) | (19.75) | (0.75) | (18.33) |

a)The average photovoltaic parameters of PSCs were obtained for 5 devices.

**Table S7.** Detailed photovoltaic properties for FA_0.79_MA_0.06_Cs_0.15_Pb(I_0.7_Br_0.3_)_3_ PSCs under different light intensity LED illumination.

| Conditions | LED  [lux] | *V*_oc_  [V] | *J*_sc_  [μA cm^-2^] | FF | Power density  [μW cm^-2^] | iPCE  [%] |
| --- | --- | --- | --- | --- | --- | --- |
| pristine TiO_2_ | 1000 | 1.03 | 125.88 | 0.73 | 94.65 | 31.67 |
|  | 800 | 1.02 | 100.89 | 0.72 | 74.09 | 30.63 |
|  | 600 | 1.01 | 76.04 | 0.71 | 54.53 | 30.06 |
|  | 400 | 0.99 | 52.45 | 0.70 | 36.35 | 29.93 |
| F-doped TiO_2_ | 1000 | 1.05 | 127.36 | 0.76 | 101.63 | 34.00 |
|  | 800 | 1.03 | 101.85 | 0.75 | 78.68 | 32.53 |
|  | 600 | 1.03 | 78.30 | 0.74 | 59.68 | 32.90 |
|  | 400 | 1.02 | 53.89 | 0.73 | 40.13 | 33.05 |

**Reference**

\[1] W. Dong, C. Zhu, C. Bai, et al., “Low-Cost Hydroxyacid Potassium Synergists as an Efficient In Situ Defect Passivator for High Performance Tin-Oxide-Based Perovskite Solar Cells,” *Angew. Chem. Int. Ed*. 62 (2023): e202302507, https://doi.org/10.1002/anie.202302507.

[2] B. Yang, J. Suo, F. Di Giacomo, et al., “Interfacial Passivation Engineering of Perovskite Solar Cells with Fill Factor over 82% and Outstanding Operational Stability on n-i-p Architecture,” *ACS Energy Lett*. 6 (2021): 3916, https://doi.org/10.1021/acsenergylett.1c01811.

[3] N. Wu, T. Yang, Z. Wang, et al., “Stabilizing Precursor Solution and Controlling Crystallization Kinetics Simultaneously for High-Performance Perovskite Solar Cells,” *Adv. Mater.* 35 (2023): 2304809, https://doi.org/10.1002/adma.202304809.

[4] Y. Li, T. Nie, X. Ren, et al., “In Situ Formation of 2D Perovskite Seeding for Record-Efficiency Indoor Perovskite Photovoltaic Devices,” *Adv. Mater*. 36 (2024): 2306870, https://doi.org/10.1002/adma.202306870.

[5] E. Han, M. Lyu, E. Choi, et al., “High-Performance Indoor Perovskite Solar Cells by Self-Suppression of Intrinsic Defects via a Facile Solvent-Engineering Strategy,” *Small* 20 (2024): 2305192, https://doi.org/10.1002/smll.202305192.

[6] C. Li, H. Sun, D. Dou, et al., “Bipolar Pseudohalide Ammonium Salts Bridged Perovskite Buried Interface toward Efficient Indoor Photovoltaics,” *Adv. Energy Mater*. 14 (2024): 2401883, https://doi.org/10.1002/aenm.202401883.

[7] K-L. Wang, H. Lu, M. Li, et al., “Ion–Dipole Interaction Enabling Highly Efficient CsPbI_3_ Perovskite Indoor Photovoltaics,” *Adv. Mater*. 35 (2023): 2210106, https://doi.org/10.1002/adma.202210106.

[8] Y. Du, Q. Tian, X. Chang, et al., “Ionic Liquid Treatment for Highest-Efficiency Ambient Printed Stable All-Inorganic CsPbI_3_ Perovskite Solar Cells,” *Adv. Mater.* 34 (2022): 2106750, https://doi.org/10.1002/adma.202106750.

[9] M. Wang, Q. Wang, J. Zhao, et al., “Low-Trap-Density CsPbX_3_ Film for High-Efficiency Indoor Photovoltaics,” *ACS Appl. Mater. Interfaces* 14 (2022): 11528, https://doi.org/10.1021/acsami.1c25207.

[10] S. Huang, S. Hou, G. Sanfo, et al., “Low-Trap-Density CsPbX_3_ Film for High-Efficiency Indoor Photovoltaics,” *Adv. Funct. Mater*. 35 (2025): 2502152, https://doi.org/10.1002/adfm.202502152.

[11] S. J. Kim, M. A. Saeed, T. H. Kim, et al., “Ultrahigh-performance indoor perovskite quantum dot photovoltaics via ligand-passivation engineering,” *Chem. Eng. J.* 488 (2024): 151154, https://doi.org/10.1016/j.cej.2024.151154.

[12] Y. S. Lee, J. Jae Do, J. W. Jung, “A comparative study of surface passivation of p-i-n perovskite solar cells by phenethylammonium iodide and 4-fluorophenethylammonium iodide for efficient and practical perovskite solar cells with long-term reliability,” *J. Alloys Compd.* 988 (2024): 174060, https://doi.org/10.1016/j.jallcom.2024.174060.

[13] N. Li, A. Feng, X. Guo, et al., “A comparative study of surface passivation of p-i-n perovskite solar cells by phenethylammonium iodide and 4-fluorophenethylammonium iodide for efficient and practical perovskite solar cells with long-term reliability,*” Adv. Energy Mater.* 12 (2022): 2103241, https://doi.org/10.1002/aenm.202103241.

[14] P. Gnanasekaran, Z-E. Shi, C. Wang, et al., “Interfacial Engineering Using C-3 Alkyl Linker-Based Carbazole-Derived SAM Layers to Achieve 41.77% Indoor Efficiency in Wide-Bandgap Perovskite Solar Cells,” *Small* 21 (2025): 2500983, https://doi.org/10.1002/smll.202500983.

[15] Z.-E. Shi, T.-H. Cheng, C.-Y. Lung, et al., “Achieving over 42 % indoor efficiency in wide-bandgap perovskite solar cells through optimized interfacial passivation and carrier transport,” *Chem. Eng. J.* 498 (2024): 155512, https://doi.org/10.1016/j.cej.2024.155512.

[16] S. Wang, M.-H. Li, Y. Zhang, et al., “Surface n-type band bending for stable inverted CsPbI_3_ perovskite solar cells with over 20% efficiency” *Energy Environ. Sci.* 16 (2023): 2572, https://doi.org/10.1039/d3ee00423f

[17] Q. Ma, Y. Wang, L. Liu, et al., “One-step dual-additive passivated wide-bandgap perovskites to realize 44.72%-efficient indoor photovoltaics,” *Energy Environ. Sci*. 17 (2024): 1637, https://doi.org/10.1039/D3EE04022D.

[18] Q. Shu, J. Xiong, B. Zhu, et al., “Constructing graded heterojunctions for rigid and flexible inverted photovoltaics under outdoor and indoor illumination,” *Chem. Eng. J*. 466 (2023): 143273, https://doi.org/10.1016/j.cej.2023.143273.
